# A Comprehensive Alanine-Scanning Mutagenesis Study Reveals Roles for Salt Bridges in the Structure and Activity of *Pseudomonas aeruginosa* Elastase

**DOI:** 10.1371/journal.pone.0121108

**Published:** 2015-03-27

**Authors:** Fei Bian, Shousong Yue, Zhenying Peng, Xiaowei Zhang, Gao Chen, Jinhui Yu, Ning Xuan, Yuping Bi

**Affiliations:** 1 Biotechnology Research Center, Shandong Academy of Agricultural Sciences, Jinan, China; 2 Shandong Provincial Key Laboratory of Crop Genetic Improvement, Ecology and Physiology, Jinan, China; 3 Shandong Crop Germplasm Center, Shandong Academy of Agricultural Sciences, Jinan, China; 4 Graduate Education Center, Shandong Academy of Agricultural Sciences, Jinan, China

## Abstract

The relationship between salt bridges and stability/enzymatic activity is unclear. We studied this relationship by systematic alanine-scanning mutation analysis using the typical M4 family metalloprotease *Pseudomonas aeruginosa* elastase (PAE, also known as pseudolysin) as a model. Structural analysis revealed seven salt bridges in the PAE structure. We constructed ten mutants for six salt bridges. Among these mutants, six (Asp189Ala, Arg179Ala, Asp201Ala, Arg205Ala, Arg245Ala and Glu249Ala) were active and four (Asp168Ala, Arg198Ala, Arg253Ala, and Arg279Ala) were inactive. Five mutants were purified, and their catalytic efficiencies (*k*_cat_/*K*_m_), half-lives (*t*_1/2_) and thermal unfolding curves were compared with those of PAE. Mutants Asp189Ala and Arg179Ala both showed decreased thermal stabilities and increased activities, suggesting that the salt bridge Asp189-Arg179 stabilizes the protein at the expense of catalytic efficiency. In contrast, mutants Asp201Ala and Arg205Ala both showed slightly increased thermal stability and slightly decreased activity, suggesting that the salt bridge Asp201-Arg205 destabilizes the protein. Mutant Glu249Ala is related to a C-terminal salt bridge network and showed both decreased thermal stability and decreased activity. Furthermore, Glu249Ala showed a thermal unfolding curve with three discernable states [the native state (N), the partially unfolded state (I) and the unfolded state (U)]. In comparison, there were only two discernable states (N and U) in the thermal unfolding curve of PAE. These results suggest that Glu249 is important for catalytic efficiency, stability and unfolding cooperativity. This study represents a systematic mutational analyses of salt bridges in the model metalloprotease PAE and provides important insights into the structure-function relationship of enzymes.

## Introduction

Metalloprotease family M4 (also known as the thermolysin family) is a very large family of zinc metalloproteases. A large number of M4 family enzymes have been implicated as virulence factors of the microorganisms that produce them [1]. For example, *Pseudomonas aeruginosa* elastase (PAE, also known as pseudolysin) is a secreted virulence factor of the opportunistic human pathogen *P*. *aeruginosa*, and vibriolysin from *Vibrio cholera* may be involved in the molecular pathogenicity of cholera. Furthermore, members of this family are important models for the study of the structure [2–5], catalytic mechanisms [6,7], and maturation [8] of metalloproteases. Comparisons of known structures of M4 family proteins have shown that members of this family possess a similar structure. For example, the mature PAE structure (PDB code: 1EZM) is composed of two domains: the N-terminal domain and the C-terminal domain [9]. The catalytic center is located in the cleft between the two domains. In the catalytic center, the single catalytic zinc ion is bonded to the protein by the side chains of three residues, including the two histidines in the HEXXH motif and a glutamic acid beyond the motif.

Members of the M4 family are found in various habitats, including thermolysin from the thermophilic bacterium *Bacillus thermoproteolyticus* [10], MCP-02 from the deep-sea bacterium *Pseudoalteromonas* sp. SM9913 [11,12], and E495 from the arctic sea-ice bacterium *Pseudoalteromonas* sp. E495 [12]. Comparisons of thermal unfolding processes indicated that mesophilic PAE has higher thermal stability than cold-adapted MCP-02 and E495. Comparative molecular dynamics (MD) studies suggested that the larger number of salt bridges in PAE might contribute to its higher thermal stability [12]. In addition to the difference in thermal stability, the comparison also revealed that PAE has a lower catalytic efficiency than MCP-02 and E495 [12]. It was suggested that optimizing the hydrogen bond persistency in MCP-02 and E495 may be an important reason for their higher catalytic efficiencies.

Salt bridges are non-covalent bonds that are important for the stability of protein structures. They are formed by negatively charged groups (usually side chains of aspartic acid or glutamic acid residues) and positively charged groups (usually side chains of lysine or arginine residues). Although structural comparisons have suggested that salt bridges may have been optimized during the cold adaptation of M4 metalloproteases [12], the contribution of salt bridges to their stability and activity has not been experimentally studied. In this study, we systematically investigated the contribution of salt bridges in PAE to its structural stability and enzymatic activity using alanine-scanning mutagenesis. The activities and thermostabilities of the mutants were compared with those of wild type PAE. This study provides insights into the structure-function relationship of the enzymes.

## Materials and Methods

### Bioinformatics

The coordinate file of PAE (code: 1EZM) [9] was downloaded from the RCSB Protein Data Bank (PDB, http://www.rcsb.org/pdb/home/home.do) [13]. The protein structure was visualized using Visual Molecular Dynamics (VMD) version 1.9 [14]. The pK_a_ values of ionizable residues were calculated using the H++ server (http://biophysics.cs.vt.edu/) [15] with the internal dielectric set at 10 and 20. The default values for the external (water) dielectric (80) and salinity (0.15 M) were used. Salt bridges were predicted based on distances (cutoff 4 Å) between positively charged oxygen atoms and negatively charged nitrogen atoms in the coordinate file 1EZM.pdb.

### Cloning of the PAE gene

The full-length PAE gene *lasB*, including the signal peptide, prosequence, and catalytic domain, was cloned using two primers as described previously [12]. The 5’ primer (GCTTGGACCATATGAAGAAGGTTTCTACGCTTGACC) contained the *NdeI* cleavage site (underlined, contains the native translational start codon of *lasB*) and the 3’ primer (CGCGCTCGAGTTACAACGCGCTCGGGCAG) contained the *XhoI* cleavage site (underlined). The amplification product was digested with the *NdeI* and *XhoI* restriction enzymes, and then cloned into the expression vector pET-22b (Novagen, USA).

### Site-directed mutagenesis

All site-directed mutations were produced using the splice-overlapping extension PCR (SOE-PCR) method. For each mutant, the 5’ portion of the gene sequence was cloned with the above mentioned 5’ primer and a specifically designed 3’ primer containing the single point mutation (Table 1). Similarly, the 3’ portion of the gene sequence was cloned with the above mentioned 3’ primer and a specifically designed 5’ primer containing the single point mutation (Table 1). The PCR fragments were purified, and the overlapping fragments were mixed together to serve as a template for the second step of the SOE-PCR. The SOE-PCR products were digested with *NdeI* and *XhoI* and ligated into the *NdeI*/*XhoI*-digested plasmid pET22b vector (Novagen). The constructed plasmids were transformed into *Escherichia coli* DH5α, and the transformants were selected and sequenced in an automated DNA sequencer model 3730 (Applied Biosystems). Then, the sequenced site-directed mutagenic plasmids were introduced into the *E*. *coli* BL21 (DE3) strain. The C-terminal local salt bridge Asp285-Arg288 was not included in this study.

**Table 1:** Primers used for site-directed mutagenesis.

Mutants	3’ Primer	5’ Primer
**Asp168Ala**	AACGAAGCGTTCTCCGCGATGGCCGGCGAGG	CTCGCCGGCCATCGCGGAGAACGCTTCGTTCATTCC
**Arg198 Ala**	GGCAGCGGTGCGCTGGCGTACATGGACCAGCCCAG	CTGGTCCATGTACGCCAGCGCACCGCTGCCC
**Asp189Ala**	TTCCTGATCGGCTACGCGATCAAGAAGGGCAGCG	GCCCTTCTTGATCGCGTAGCCGATCAGGAAGTCGTTC
**Arg179Ala**	GCCGAGTTCTACATGGCGGGCAAGAACGACTTCCTG	GTCGTTCTTGCCCGCCATGTAGAACTCGGCAGCCTC
**Asp201Ala**	GCGCTGCGCTACATGGCGCAGCCCAGCCGCGAC	GCGGCTGGGCTGCGCCATGTAGCGCAGCGCACCG
**Arg205Ala**	GACCAGCCCAGCGCGGACGGGCGATCCATC	GGATCGCCCGTCCGCGCTGGGCTGGTCCATG
**Arg245Ala**	CCGGGCTGGGATACCGCGAAGGCCTTCGAGGTGTTC	CACCTCGAAGGCCTTCGCGGTATCCCAGCCCGGC
**Asp253Ala**	TTCGAGGTGTTCGTCGCGGCCAACCGCTACTACTG	GTAGTAGCGGTTGGCCGCGACGAACACCTCGAAGG
**Glu249Ala**	ACCCGCAAGGCCTTCGCGGTGTTCGTCGACGCC	GGCGTCGACGAACACCGCGAAGGCCTTGCGGG
**Arg279Ala**	CGCTCGGCGCAGAACGCGAACTACTCGGCGGCTG	AGCCGCCGAGTAGTTCGCGTTCTGCGCCGAGC

### Protein expression and purification

The *E*. *coli* BL21 (DE3) strains harboring the target genes were cultured to an OD_600_ of approximately 1.0, and then induced with 1 mM isopropyl 1-thio-β-D-galactopyranoside (IPTG) at 37°C overnight. The cells were harvested by centrifugation at 8000 rpm for 5 min. After ultrasonication in 50 mM Tris-HCl buffer (pH 8.0), the samples were centrifuged at 11,000 rpm for 15 min; then, the supernatants were incubated at 30°C for 2~24 h. Their hydrolytic activities were detected using casein as a substrate [11]. The precipitants were dissolved in 50 mM Tris-HCl buffer containing 8 M urea and analyzed by 12.5% SDS-PAGE. The *E*. *coli* BL21 (DE3) strain containing the empty plasmid pET22b vector was constructed as a control and treated with the same procedure.

PAE and the active mutants were purified as described previously [12]. After ultrasonication and centrifugation, the supernatant was precipitated with 60% solid ammonium sulfate. After dialysis in 50 mM Tris-HCl buffer (pH 9.5), the crude proteins were separated using a DEAE-Sepharose Fast Flow column (Amersham Biosciences) with a linear NaCl gradient from 0 to 0.5 M in 50 mM Tris-HCl buffer (pH 9.5). The protease concentrations were measured using the bicinchoninic acid (BCA) method with bovine serum albumin as a standard. Purified proteases were dialyzed against 50 mM Tris-HCl buffer (pH 8.0) to remove NaCl and stored at -20°C.

### Enzymatic activity measurement

The enzymatic activities of the PAE and mutant culture supernatants were assessed with casein as the substrate at 55°C in 50 mM Tris-HCl buffer (pH 7.5) [11]. For the determination of thermostability at 60°C, the enzymes were incubated at 60°C for 15–150 min and then immediately placed on ice. The residual activities were determined at 55°C. The half-lives (*t*_1/2_) were determined by linear fitting using the equation ln(*A*/*A*_0_) = -0.693 / *t*_1/2_ * *t*, where *A* is the residual activity at incubation time *t* and *A*_0_ is the initial activity (*t* = 0).

Specific constant *k*_cat_/*K*_m_ values toward the dipeptide substrate 3-(2-furylacryloyl)glycyl-L-leucine amide (FAGLA) were determined as previously described [12]. Generally, the decrease in absorbance at 345 nm (Δε_345nm_ = 317 M^-1^cm^-1^) was monitored every 1 s for 10 min using a Jasco V-550 spectrophotometer at 25°C. *k*_cat_/*K*_m_ was determined using the relationship *k*_cat_/*K*_m_ = *v*_0_/*S*_0_/*E*_0_, which is valid at *S*_0_<<*K*_m_. FAGLA was purchased from Bachem AG (Bubendorf, Switzerland), and its concentration was determined spectrophotometrically using ε_345nm_ = 766 M^-1^cm^-1^ [16]. The initial substrate concentration *S*_0_ was 0.5 mM, and the initial enzyme concentration *E*_0_ was 0.09–0.66 μM, depending on the activity of PAE and the mutants. The temperature was kept constant using a thermostat (Julabo, Germany).

### Circular dichroism (CD) measurements

The thermal unfolding curves were recorded using a Chirascan-plus Circular Dichroism spectrometer (Applied Photophysics, UK) equipped with a computer-controlled thermostat. The signal was recorded at 222 nm with a bandwidth of 1 nm. The temperature was monitored using an internal sensor, and the heating rate was 1.0°C per min. A 0.1-cm path length cell was used. The protein concentrations were 0.2–0.4 mg/ml. Tris-HCl buffer (50 mM, pH 7.5) was used. The fraction of unfolded protein (*f*_U_) was calculated using the equation *f*_U_ = (*CD*_T_—*CD*_40_) / (*CD*_80_—*CD*_40_), where *CD*_T_ is the signal at temperature *T*, *CD*_40_ is the signal at 40°C and *CD*_80_ is the signal at 80°C. The CD signal at 40°C (*CD*_40_) was used to represent the native state (N, *f*_U_ = 0), and the signal at 80°C (*CD*_80_) was used to represent the unfolded state (U, *f*_U_ = 1). The apparent melting temperature *T*_m_ was estimated as the temperature of *f*_U_ = 0.5.

## Results

### Salt bridges in PAE

**Fig 1:**
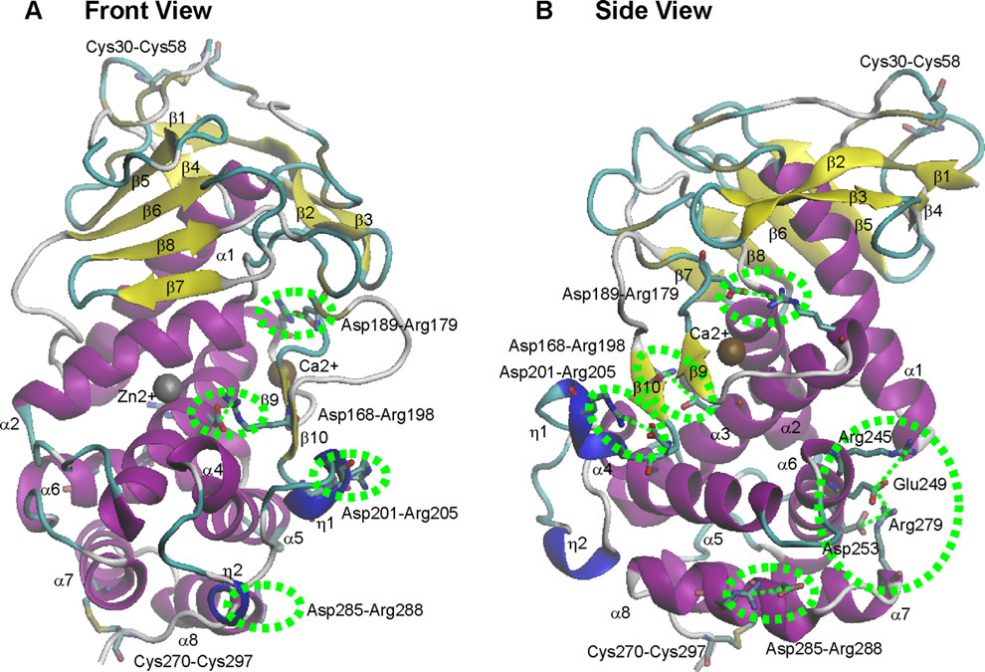
Salt bridges in PAE. (A) Front view. (B) Side view. Salt bridges are indicated using green circles.

The pK_a_ values of ionizable residues were calculated for all of the ionizable residues in PAE (coordinate file, 1EZM.pdb) with the H++ server [15]. The calculation showed that all of the histidine residues in PAE have pK_a_ values lower than 7.5 (Table 2). Because PAE activity is optimal at pH 7.5–8.0 [12], all histidine residues were assumed to be neutral and thus were excluded from the salt bridge calculation. Based on the distances (cutoff 4 Å) between positively charged oxygen atoms and negatively charged nitrogen atoms, seven salt bridges were found in PAE (coordinate file, 1EZM.pdb), including Asp168-Arg198, Asp189-Arg179, Asp201-Arg205, Glu249-Arg245, Glu249-Arg279, Asp253-Arg279, and Asp285-Arg288 (Fig. 1).

**Table 2:** Predicted pK_a_ values for histidines in PAE by the H++ server.

Residues	pK_a_ [ε (internal) = 10]	pK_a_ [ε (internal) = 20]
**His77**	7.059	7.139
**His98**	6.833	6.953
**His105**	5.502	6.406
**His140**	<0	<0
**His144**	<0	<0
**His223**	7.409	7.248
**His224**	6.749	6.864

Salt bridge Asp168-Arg198 is located in very close proximity to the active site, with Asp168 on α-helix α3 and Arg198 on β-strand β10. Salt bridge Asp189-Arg179 is adjacent to the Ca^2+^ ion and relatively distant from the active site, with Arg179 on α3 and Asp189 on the loop between β9 and β10. Salt bridge Asp201-Arg205 is also relatively distant from the active site, with Arg205 on 3_10_-helix η1 and Asp201 on the adjacent loop between β10 and 3_10_-helix η1. Three salt bridges are located in the C-terminal domain and form a salt bridge network Arg245-Glu249-Arg279-Asp253. Arg279 is located on the C-terminus of α-helix α7, whereas the other three residues are located on α-helix α6. Therefore, α-helices α6 and α7 are combined by this salt bridge network. Salt bridge Asp285-Arg288 is a local salt bridge located in the C-terminal α-helix α8.

### Construction and expression of mutants

To evaluate the contribution of the salt bridges to the stability and activity of PAE, an alanine-scanning mutagenesis study was performed for six salt bridges, including Asp168-Arg198, Asp189-Arg179, Asp201-Arg205, Glu249-Arg245, Glu249-Arg279, and Asp253-Arg279 (Fig. 1). A total of ten mutants were constructed, including Asp168Ala, Arg198Ala, Asp189Ala, Arg179Ala, Asp201Ala, Arg205Ala, Arg245Ala, Glu249Ala, Arg279Ala, and Asp253Ala. All mutants were expressed in *E*. *coli*.

**Fig 2:**
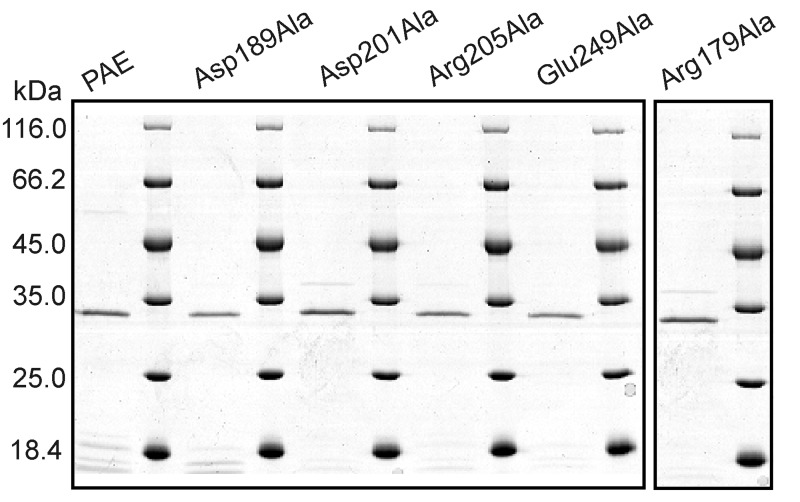
SDS-PAGE analysis of the purified PAE and mutants. Mutant Arg179Ala was purified after a 12-h incubation. The other mutants were purified after a 2-h incubation.

The auto-cleavage of the precursor that removes the prosequence is a required step for the activation of PAE proteolytic activity. After ultrasonication and centrifugation, the supernatants were incubated at 30°C to allow the auto-maturation of the enzymes. The activities were assessed with casein as the substrate. Activities were detected for PAE and mutants Asp189Ala, Asp201Ala, Arg205Ala, and Glu249Ala after a 2-h incubation. When the incubation time was extended to 12 h, activities were detected for mutants Arg179Ala and Arg245Ala. Activities were not observed for mutants Asp168Ala, Arg198Ala, Arg279Ala, or Asp253Ala even after a 24-h incubation. Therefore, PAE and mutants Asp189Ala, Arg179Ala, Asp201Ala, Arg205Ala, and Glu249Ala were purified (Fig. 2). Arg245Ala was not purified because the amount of the active mature form was too low.

### Comparison of activities

**Fig 3:**
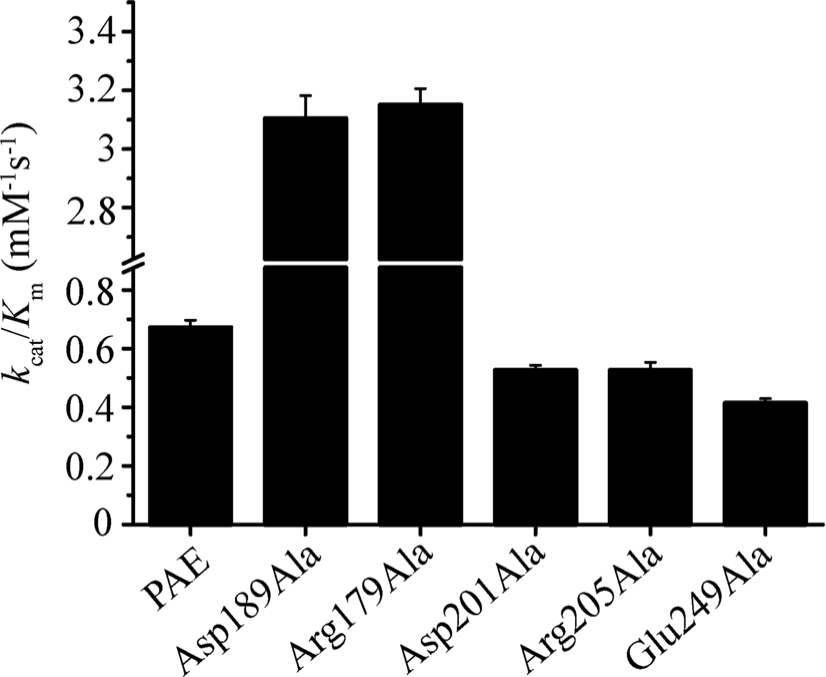
Specific constant *k*_cat_/*K*_m_ values of PAE and the active mutants. The *k*_cat_/*K*_m_ values were measured at 25°C with FAGLA as the substrate. Mutant Arg179Ala was purified after a 12-h incubation. The other mutants were purified after a 2-h incubation. Each measurement was repeated three times, and all of the standard deviations were within 5% of the corresponding mean values.

Catalytic efficiencies (defined as the constant *k*_cat_/*K*_m_) of the recombinant PAE and the purified active mutants were measured at 25°C using the synthetic peptide FAGLA as the substrate (Fig. 3). Recombinant PAE had a catalytic efficiency of 0.672 mM^-1^s^-1^. In comparison, mutants Asp201Ala, Arg205Ala and Glu249Ala had decreased catalytic efficiencies (0.526, 0.526, and 0.414 mM^-1^s^-1^, respectively). Different from the above three mutants, mutants Asp189Ala and Arg179Ala had elevated catalytic efficiencies (3.10 and 3.15 mM^-1^s^-1^, respectively). Both mutants Asp201Ala and Arg205Ala had decreased catalytic efficiencies, suggesting that the existence of salt bridge Asp201-Arg205 promotes catalytic efficiency. Similarly, the existence of salt bridge Asp189-Arg179 most likely decreases the catalytic efficiency.

### Comparison of the half-lives

**Fig 4:**
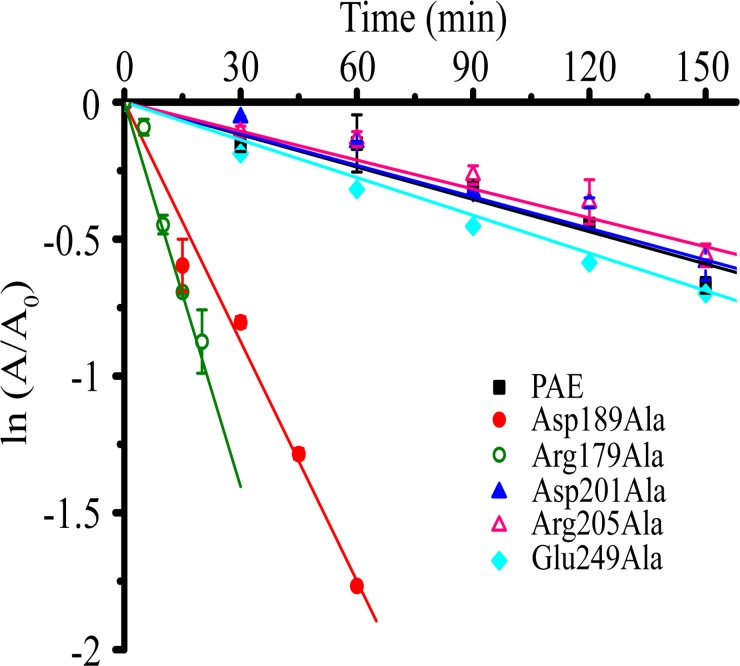
Thermal inactivation of PAE and the active mutants at 60°C. The half-life *t*_1/2_ was calculated by linear fitting using equation ln (*A*/*A*_0_) = -0.693/*t*_1/2_ * *t*. The vertical axis shows the natural logarithm of the residual activity [ln (*A*/*A*_0_)]. The half-life *t*_1/2_ corresponds to the point (*t*_1/2_, -0.693) on the fitted line.

A previous study showed that PAE had an optimum temperature of ~62°C [12]. Therefore, the half-lives (*t*_1/2_) of PAE and its mutants were measured at 60°C to gain insights into the relationship between the salt bridges and the half-life (Fig. 4). PAE had a half-life of 176 min. In contrast, the half-lives of the mutants Asp189Ala and Arg179Ala were only 24 min and 12 min, respectively. The dramatically decreased half-lives of these two mutants suggest that the salt bridge Asp189-Arg179 is important for the kinetic stability of PAE. The half-life of mutant Glu249Ala was also reduced compared with PAE (110 min versus 176 min). Mutants Asp201Ala and Arg205Ala had slightly longer half-lives than PAE (180 min and 178 min, respectively), suggesting that the kinetic stability is compromised in PAE due to the existence of the salt bridge Asp201-Arg205.

### Thermal unfolding

**Fig 5:**
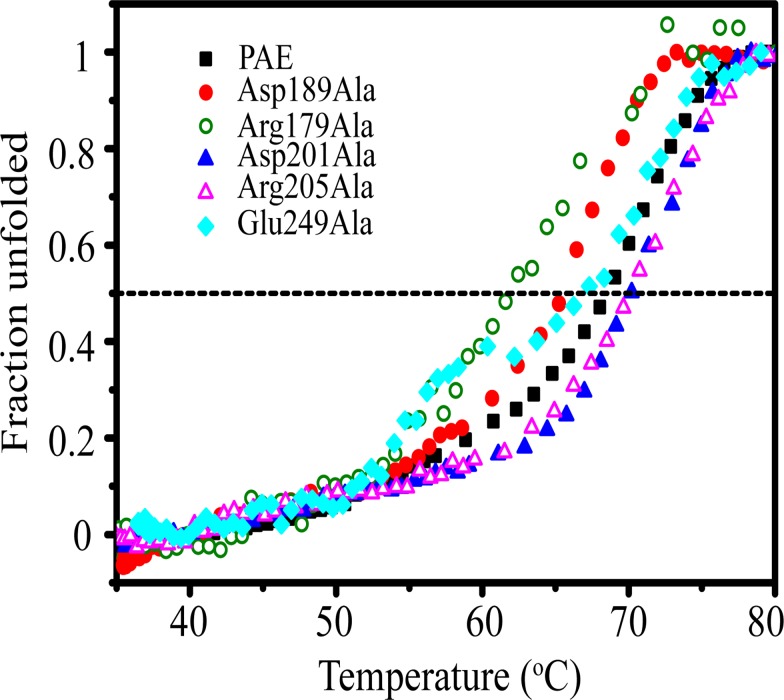
Thermal unfolding of PAE and the active mutants. The dashed line indicates an unfolded fraction of 0.5.

The thermostabilities of the mutants and recombinant PAE were assessed by monitoring the thermal denaturation process using CD at 222 nm. For PAE and the mutants, the CD signal at 222 nm increased slowly as the temperature increased from 35°C to 50°C and reached its maximum at 75–80°C. The unfolding curve was scaled with the CD value at 40°C as the native state (*f*_U_ = 0) and the CD value at 80°C as the unfolded state (*f*_U_ = 1) (Fig. 5). The wild type enzyme had a *T*_m_ value of ~69°C. Mutant Asp189Ala had a decreased *T*_m_ value of ~65°C, which is consistent with its decreased half-life (24 min). Mutant Arg179Ala had a dramatically decreased *T*_m_ value (~62°C), which is consistent with its dramatically decreased half-life (12 min). Both Asp201Ala and Arg205Ala had a slightly increased *T*_m_ value of ~70°C, which is consistent with their slightly increased half-lives (180 min and 178 min).

Compared with PAE and the other mutants, mutant Glu249Ala displayed a different unfolding curve (Fig. 5). PAE and the mutants Asp189Ala, Arg179Ala, Asp201Ala, and Arg205Ala unfolded rapidly in the range from ~60°C to ~75°C; thus, the unfolding could be modeled as a two-state process, proceeding from the native state (N) to the unfolded state (U). For Glu249Ala, in addition to N and U a metastable, partially unfolded state (I) was discernable in the unfolding curve at 58–62°C. Accordingly, the thermal unfolding process can be modeled as a three-state process: N → I → U. The N → I transition occurred at approximately 50–56°C, and the I → U transition occurred at approximately 63–75°C. The existence of state I suggests a low unfolding cooperativity for Glu249Ala. Therefore, the single point mutation Arg249Ala resulted in not only a decrease in the catalytic efficiency and thermal stability but also in changes in the unfolding process.

## Discussion

### Insights into the salt bridge-stability-activity relationship

A comparison of psychrophilic and/or cold-adapted enzymes with their mesophilic counterparts indicates that the psychrophilic/cold-adapted enzymes usually have higher activities at low temperatures and lower stabilities at high temperatures [17–20]. It has been suggested that psychrophilic/cold-adapted enzymes achieve higher activity by increasing their structural flexibility, but this change occurs at the expense of their structural stability [17–20]. Enzymes may adopt different strategies for cold adaptation, among which decreasing salt bridge numbers and/or hydrogen bond numbers have been adopted by many different enzymes [17–20]. However, how the decrease in salt bridge and hydrogen bond numbers affects the enzyme flexibility and activity is not well understood. In this study, the contribution of salt bridges to thermostability and activity was studied using PAE as a model.

Among the ten mutants constructed, only six showed activities; five of these mutants were successfully purified. Both mutants related to salt bridge Asp189-Arg179 showed decreased stabilities (as represented by *T*_m_ and *t*_1/2_) and increased activities towards the synthetic substrate FAGLA. These results suggest that the salt bridge Asp189-Arg179 plays an important role in the stabilization of the PAE structure at the expense of its activity. The case for salt bridge Asp201-Arg205 is different. The two mutants related to this salt bridge showed slightly increased stabilities (as represented by *T*_m_ and *t*_1/2_) and slightly decreased activities. Therefore, the salt bridge Asp201-Arg205 slightly destabilizes the structure.

Although the above results indicated that salt bridges Asp189Ala-Arg179 and Asp201-Arg205 have different contributions to stability and activity, the stability-activity relationship suggested by these results generally is consistent with the hypothesis presented in previous studies that higher activity is usually related to lower stability, and vice versa [17–20]. However, the mutant Glu249Ala does not follow the above stability-activity relationship, because this mutant showed both decreased stability and activity.

### Correlation between salt bridge persistency and mutant activity

In the previous study, MD simulations were used to study the persistency (the ratio of the time when the bond exists in the protein structure, calculated based on the MD simulation trajectories) of salt bridges and hydrogen bonds in PAE and close relatives [12]. It was shown that salt bridges Asp168-Arg198 and Asp253-Arg279 have a persistency of 100% at both 280 K and 310 K [12]. This result indicates that these two salt bridges exist in all frames of the MD simulation trajectories. The high persistency of 100% suggests that the salt bridge is very important for the protein structure. Other salt bridges, including Asp189-Arg179, Asp201-Arg205, Glu249-Arg245, Glu249-Arg279 and Asp285-Arg288, had a persistency lower than 100% at 280 K or 310 K [12]. This result indicates that these salt bridges do not exist in all frames of the MD simulations, and therefore should be less important than those with a persistency of 100%. In this study, we evaluated the contributions of these salt bridges to the structure using alanine-scanning mutagenesis. Our results showed that for salt bridges with a persistency of 100% (i.e., Asp168-Arg198 and Asp253-Arg279), the single point mutations to related residues resulted in inactive mutants (Asp168Ala, Arg198Ala, Arg279Ala, and Asp253Ala). For salt bridges with a persistency of <100%, the single point mutations to related residues resulted in six active mutants (Asp189Ala, Arg179Ala, Asp201Ala, Arg205Ala, Arg245Ala and Glu249Ala). Therefore, a persistency of 100% indicates a strong salt bridge, and disrupting such a strong salt bridge leads to inactive mutants.

### Expression and maturation of mutants

**Fig 6:**
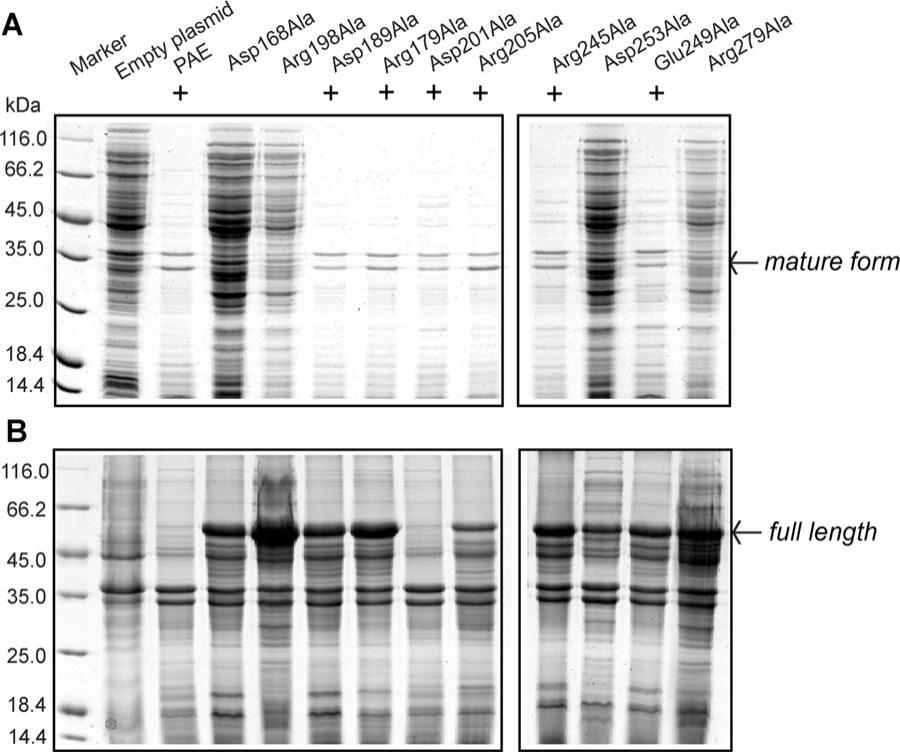
SDS-PAGE analysis of supernatants incubated for maturation (A) and precipitates (B) after ultrasonication. For PAE and all mutants, the same purification and maturation procedure (with the exception of different incubation times, 2 h or 12 h) were applied, and the same amounts (in volume) of the samples were loaded into different lanes of the SDS-PAGE.

Only six out of ten mutants showed activities. SDS-PAGE analysis of the incubated (for 2 or 12 h) supernatants after ultrasonication indicated that the lanes for PAE and the active mutants contained less protein bands and lower amounts of proteins than the inactive mutants (Fig. 6A). However, due to the overlap of bands for the mature target enzymes and the protein products of the empty plasmid (Fig. 6A), it is unclear whether the inactive mutants were expressed. Therefore, we also analyzed the precipitates after ultrasonication with SDS-PAGE. The lanes for most mutants, including all of the inactive mutants, contained a clear band representing the full-length precursor of the protein (Fig. 6B, 498 aa, ~54 kDa). Therefore, all of the inactive mutants were expressed.

The full length precursor of PAE contains an N-terminal presequence that is not included in the mature enzyme. For PAE, mature (active) enzymes without this presequence can be obtained after a 2-h incubation. Four of the mutants showed activities after a 2-h incubation, whereas another two showed activities after a 12-h incubation. The different incubation times indicate that disrupting salt bridges may also affect the auto-maturation process.
